# The Lectin from *Schinus terebinthifolia* Raddi Leaves (SteLL) Exhibited Anti-Inflammatory Activity in Lipopolysaccharide-Induced Acute Lung Injury in Mice

**DOI:** 10.3390/ijms27083394

**Published:** 2026-04-10

**Authors:** Amanda de Oliveira Marinho, Maria Nívea Bezerra da Silva, Ana Karollina Viana Chagas, Alícia Natalie Silva dos Santos, Pedro Paulo Marcelino Neto, Patrícia Maria Guedes Paiva, Emmanuel Viana Pontual, Anderson Arnaldo da Silva, Leydianne Leite de Siqueira Patriota, Thiago Henrique Napoleão

**Affiliations:** 1 Departamento de Bioquímica, Centro de Biociências, Universidade Federal de Pernambuco, Recife 50670-901, PE, Brazil; amanda.marinho@ufpe.br (A.d.O.M.); maria.nivea@ufpe.br (M.N.B.d.S.); karollina.viana@ufpe.br (A.K.V.C.); alicia.natalie@ufpe.br (A.N.S.d.S.); pedro.paulo87@hotmail.com (P.P.M.N.); leydianne.patriota@ufpe.br (L.L.d.S.P.); thiago.napoleao@ufpe.br (T.H.N.); 2 Departamento de Morfologia e Fisiologia Animal, Universidade Federal Rural de Pernambuco, Recife 52171-900, PE, Brazil; emmanuel.pontual@ufrpe.br; 3 Departamento de Anatomia, Centro de Biociências, Universidade Federal de Pernambuco, Recife 50670-901, PE, Brazil; anderson.arnaldo@ufpe.br

**Keywords:** *Schinus terebinthifolia*, plant lectins, respiratory disease

## Abstract

Acute lung injury (ALI) is a severe inflammatory condition associated with high morbidity and mortality, and there are currently no specific pharmacological treatments available. In this context, plants and natural products have emerged as promising therapeutic alternatives. SteLL (*Schinus terebinthifolia* Raddi leaf lectin) has demonstrated several biological activities, including anti-inflammatory and immunomodulatory effects. This study evaluated the anti-inflammatory effects of SteLL in a murine model of lipopolysaccharide (LPS)-induced ALI. Female BALB/c mice received intraperitoneal (i.p.) administration of SteLL (1, 5, or 10 mg/kg), dexamethasone (2 mg/kg), or vehicle (PBS). Sixty minutes later, ALI was induced by intranasal instillation of 25 µL of LPS (1 μg/μL). After 24 h, the animals were euthanized. Bronchoalveolar lavage fluid (BALF) was obtained to evaluate inflammatory parameters and lungs were collected for histopathological analysis. The tested doses of SteLL resulted in a 45–66% lower leukocyte infiltration. The group treated with 5 mg/kg exhibited a lower proportion of neutrophils and a higher proportion of mononucleated cells. Pre-treatment with SteLL also minimized plasma leakage and myeloperoxidase (MPO) activity. Furthermore, SteLL attenuated the release of pro-inflammatory cytokines at all tested doses as well as prevented nitric oxide (NO) production at the highest dose (10 mg/kg). Histopathological analysis showed that SteLL (5 and 10 mg/kg) attenuated LPS-induced lung injury. Overall, SteLL demonstrated significant anti-inflammatory effects, showing its potential as a plant-derived compound for modulating pulmonary inflammation.

## 1. Introduction

Acute lung injury (ALI) is a condition of respiratory failure resulting from multifactorial damage to the alveolar epithelium. The underlying inflammatory process is characterized by the migration of leukocytes to the pulmonary parenchyma, accumulation of interstitial fluid, and increased release of pro-inflammatory mediators such as cytokines and nitric oxide (NO), as well as the formation of reactive oxygen species mediated by myeloperoxidase (MPO) [1,2,3,4]. Acute respiratory distress syndrome (ARDS) represents the most severe clinical manifestation of ALI and constitutes a significant clinical challenge due to its rapid progression and high rates of morbidity and mortality [4,5,6].

It is estimated that approximately 3 million people worldwide are affected by ALI each year [7,8,9]. ALI generally has an infectious origin, with respiratory viruses and Gram-negative bacteria being particularly prominent. During the COVID-19 pandemic, the SARS-CoV-2 virus emerged as a new trigger for ALI and its most severe outcome, ARDS, contributing significantly to an exorbitant increase in mortality and morbidity rates among patients affected by the disease [6,10]. However, although SARS-CoV-2 drew global attention to viral causes of ALI, Gram-negative bacteria such as *Escherichia coli* and *Klebsiella pneumoniae* have long represented a major and persistent threat. This is largely due to lipopolysaccharide (LPS), a structural endotoxin of their outer membrane that has been recognized for decades as a potent inducer of ALI [11,12].

Current conventional therapies for ALI and ARDS primarily consist of mechanical ventilatory support and the administration of anti-inflammatory drugs, such as corticosteroids, vasodilators, and modulators of the inflammatory cascade [13,14,15,16]. Despite these interventions, hospital mortality rates can reach 40%, representing a significant clinical challenge [17,18].

*Schinus terebinthifolia* Raddi (Anacardiaceae), popularly known as aroeira or Brazilian pepper tree, has widespread use in traditional medicine. Due to its anti-inflammatory, antipyretic, analgesic, and depurative activities, it is used in the treatment of urinary tract infections, gastrointestinal disorders, and other health conditions [19,20,21]. Leaf preparations are popularly used as anti-inflammatory agents [22], relieving conditions such as bronchitis, ulcers, and wounds [23]. Among the bioactive constituents of *S. terebinthifolia* leaves, there is a lectin called SteLL (*S. terebinthifolia* leaf lectin) [24].

Lectins are carbohydrate-binding proteins that can selectively and reversibly bind to carbohydrates [25], exhibiting distinct biological activities, including antibacterial [26], antifungal [27], antibiofilm [28], nematicidal [29], and insecticidal [30] effects, as well as pharmacological properties such as antinociceptive, anti-inflammatory, and antitumor activities [31]. SteLL is a glycoprotein with a molecular mass of 12.4 kDa and hemagglutinating activity that is resistant to variations in pH (5.0–8.0) and temperature (up to 100 °C), as well as exhibiting functional stability in the presence of Ca^2+^ and Mg^2+^ ions [24,32]. The safety of SteLL administered intraperitoneally or orally at a single dose of 100 mg/kg was demonstrated in mice [33]. Studies have already demonstrated in vitro antimicrobial [24] and immunomodulatory [32] effects of SteLL. In vivo, SteLL has shown antitumor activity against sarcoma 180 [34], anti-infective effects [35], antidepressant and anxiolytic effects in different contexts [36,37], and antinociceptive activity [38,39], all in mice.

Marinho et al. [33] reported that SteLL demonstrated significant anti-inflammatory activity in acute inflammation models, including peritonitis and paw edema, both induced by carrageenan in mice. The results indicated the modulatory potential of SteLL on cellular inflammatory responses and edematous processes, as evidenced by reductions in leukocyte migration, MPO activity, protein extravasation, and the levels of NO and pro-inflammatory cytokines.

The traditional use of *S. terebinthifolia* leaves in respiratory inflammatory conditions suggests the presence of bioactive compounds capable of modulating pulmonary inflammatory responses. In addition, the diverse biological activities described for SteLL highlight its broad pharmacological potential, including an ability to modulate inflammatory processes. Therefore, this study aimed to investigate the preventive effects of SteLL on the pulmonary inflammatory response in an experimental model of LPS-induced ALI.

## 2. Results

SteLL was successfully purified following the original protocol described by Gomes et al. [24], and the batch used in this study showed functional activity, with a specific hemagglutinating activity of 6336, indicating the presence of active SteLL molecules.

**Figure 1:**
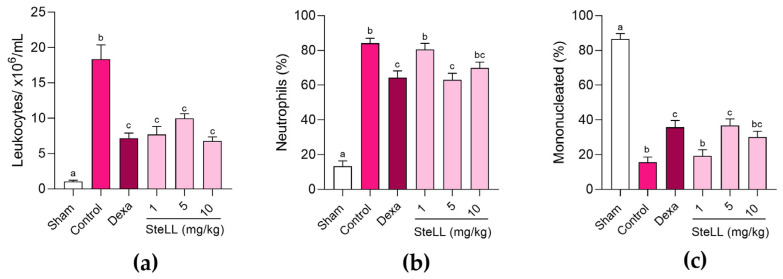
Evaluation of leukocyte migration in the bronchoalveolar fluid of healthy mice (Sham) and those subjected to acute lung inflammation induced by lipopolysaccharide and previously treated intraperitoneally with PBS (control), SteLL (1, 5, and 10 mg/kg), or dexamethasone (Dexa, 2 mg/kg). (**a**) Leukocyte count. (**b**) Percentage of neutrophils. (**c**) Percentage of mononucleated cells. The results are expressed as mean ± standard error of the mean (SEM) (*n* = 8 per group). Different letters indicate significant differences (*p* < 0.05) between the groups, based on analysis of variance (ANOVA) followed by Tukey’s test.

Figure 1a shows the total leukocyte counts in the bronchoalveolar lavage fluid (BALF) of healthy (Sham) mice and of animals with LPS-induced ALI previously treated with SteLL (1, 5, or 10 mg/kg), dexamethasone (2 mg/kg), or vehicle (phosphate-buffered saline, PBS; control), revealing significant differences between the groups (F_5,42_ = 29.13; *p* < 0.0001). The control group exhibited markedly higher (*p* < 0.0001) leukocyte migration (18,360 ± 2020 cells/mL) compared with the Sham group (1025 ± 190 cells/mL), confirming the validity of the experimental model. SteLL-treated groups demonstrated an anti-inflammatory effect, with leukocyte migration 58.07 ± 6.15% (1 mg/kg), 45.55 ± 3.48% (5 mg/kg), and 63.11 ± 3.11% (10 mg/kg) lower than in the control group (*p* < 0.0001). There were no significant differences (*p* > 0.05) among the groups treated with distinct doses of SteLL. Dexamethasone-treated group showed leukocyte migration that was 60.86 ± 3.81% lower than the control group (*p* < 0.0001) but similar (*p* > 0.05) to SteLL treatments.

Differential leukocyte counts in the BALF was performed to evaluate the proportion of neutrophils (Figure 1b; F_5,42_ = 53.87; *p* < 0.0001) and mononucleated cells (Figure 1c; F_5,42_ = 53.82; *p* < 0.0001). SteLL at 5 mg/kg resulted in a significantly (*p* = 0.0015) lower percentage of neutrophils (63.13 ± 3.74%) compared with the control group (84.13 ± 2.94%), whereas the other doses did not significantly reduce (*p* > 0.05) neutrophil counts compared to the control group (Figure 1b). Correspondingly, the group pre-treated with SteLL at 5 mg/kg showed higher (*p* = 0.0013) proportion of mononucleated cells (36.88 ± 3.74%) than in control (15.63 ± 2.96%) (Figure 1c). Dexamethasone group also showed lower (*p* = 0.0029) neutrophil proportion (64.25 ± 3.98%) (Figure 1b) and higher (*p* = 0.0026) mononucleated cells proportion (35.75 ± 3.98%) (Figure 1c) relative to the control group, while showed an effect similar (*p* > 0.999) to that of SteLL at 5 mg/kg.

**Figure 2:**
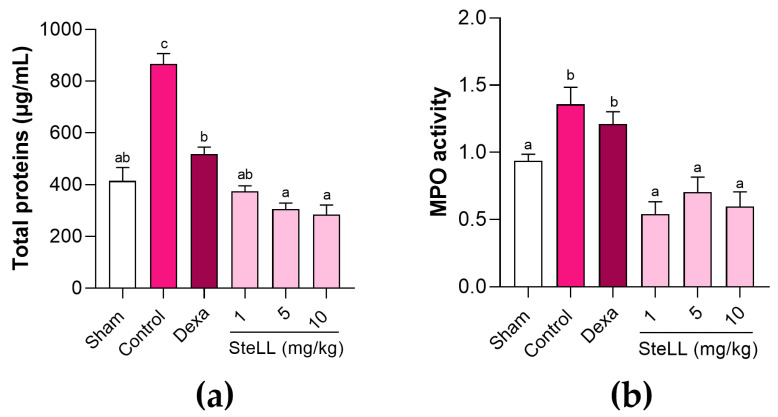
Total protein levels (µg/mL) (**a**) and myeloperoxidase (MPO) activity (**b**), in the bronchoalveolar fluid of healthy mice (Sham) and those subjected to acute lung inflammation induced by lipopolysaccharide and previously treated intraperitoneally with PBS (control), SteLL (1, 5, and 10 mg/kg), or dexamethasone (Dexa, 2 mg/kg). Results are expressed as mean ± standard error of the mean (SEM) (*n* = 8 per group). Different letters indicate significant differences (*p* < 0.05) between the groups, based on analysis of variance (ANOVA) followed by Tukey’s test.

Plasma extravasation into the BALF was evaluated by assessing the total protein content (Figure 2a; F_5,42_ = 38.03; *p* < 0.0001). In comparison with control group, the animals pre-treated with SteLL (1, 5, and 10 mg/kg) showed significantly less (*p* < 0.0001) plasma extravasation into the BALF, being the values 57.36 ± 2.48%, 65.12 ± 2.69%, and 67.57 ± 4.26% lower, respectively. In turn, dexamethasone treatment led to plasma extravasation 40.85 ± 3.07% lower than in control (*p* < 0.0001). The data also showed that total protein content in the SteLL- and dexamethasone-treated groups was similar (*p* > 0.05) to that in the BALF of healthy (Sham) animals, whereas SteLL at 5 and 10 mg/kg resulted in lower protein content (*p* < 0.001) compared with the dexamethasone group.

Figure 2b shows the MPO activity in the BALF of the studied groups (F_5,42_ = 9.241; *p* < 0.0001). Treatment with SteLL (1, 5, and 10 mg/kg) also led to lower (*p* ≤ 0.0005) MPO activity in the BALF compared with the negative control group, differently of dexamethasone (*p* = 0.8960). SteLL doses showed similar levels (*p* > 0.85) of MPO activity, with enzyme activity values equivalent (*p* > 0.05) to the healthy (Sham) group.

**Figure 3:**
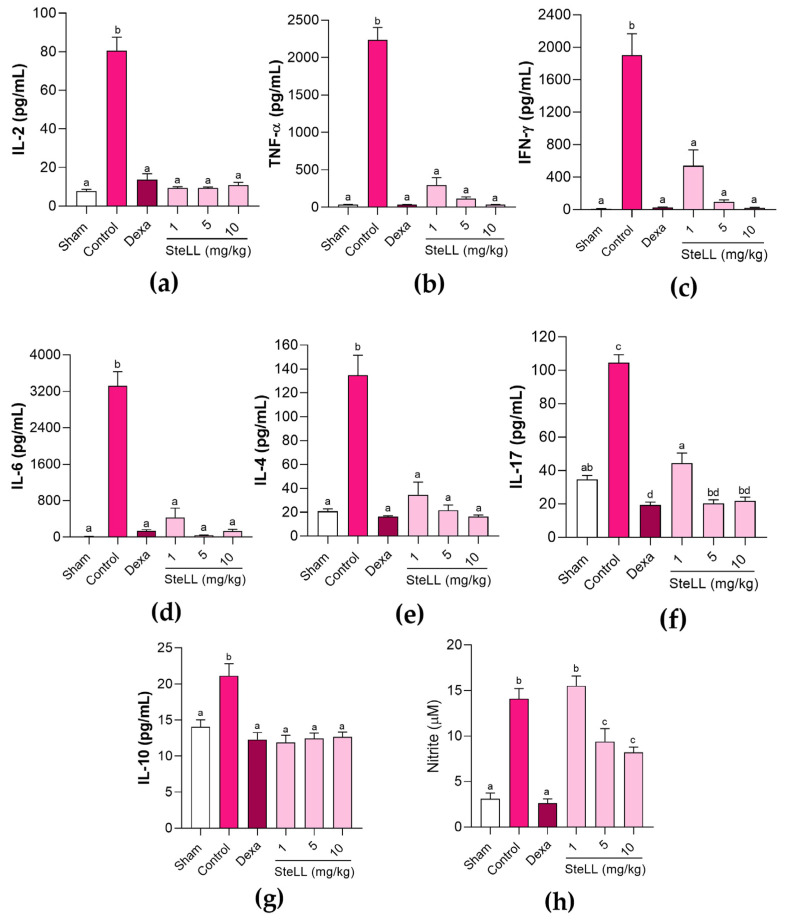
Cytokines (**a**–**g**) and nitric oxide (**h**) levels in the bronchoalveolar fluid of healthy mice (Sham) and those subjected to acute lung inflammation induced by lipopolysaccharide and previously treated intraperitoneally with PBS (control), SteLL (1, 5, and 10 mg/kg), or dexamethasone (Dexa, 2 mg/kg). Bars represent the mean ± standard error of the mean (SEM) of data from animals (*n* = 8 per group). Different letters indicate significant differences (*p* < 0.0001) between the groups, based on analysis of variance (ANOVA) followed by Tukey’s test and false discovery rate (FDR) correction.

Significant differences were detected for the levels of the interleukin (IL) 2 (Figure 3a, F_5,42_ = 80.81; *p* < 0.0001), tumor necrosis factor (TNF) α (Figure 3b, F_5,42_ = 123.6; *p* < 0.0001), interferon (IFN)-γ (Figure 3c, F_5,42_ = 31.35; *p* < 0.0001), IL-6 (Figure 3d, F_5,42_ = 39.70; *p* < 0.0001), IL-4 (Figure 3e, F_5,42_ = 31.31; *p* < 0.0001), IL-17 (Figure 3f, F_5,42_ = 86.73; *p* < 0.0001) and IL-10 (Figure 3g, F_5,42_ = 11.10; *p* < 0.0001) in BALF among the different groups. The LPS administration was highly immunogenic, causing a significant increase in all measured cytokines in the control group compared with the Sham group. Pre-treatment with SteLL (1, 5, and 10 mg/kg) or dexamethasone (2 mg/kg) resulted in significantly lower (*p* < 0.0001) levels of both pro-inflammatory (IL-2, TNF-α, IFN-γ, IL-6, and IL-17) and anti-inflammatory (IL-4 and IL-10) cytokines. There were significant differences among SteLL doses only for IL-17, with the animals that received the lowest dose (1 mg/kg) showing a higher level (*p* ≤ 0.0006) of this cytokine in the BALF (Figure 3f). In addition, SteLL at 5 and 10 mg/kg and dexamethasone groups showed lower NO levels relative to the control group (Figure 3h, F_5,42_ = 32.11; *p* < 0.0001).

**Figure 4:**
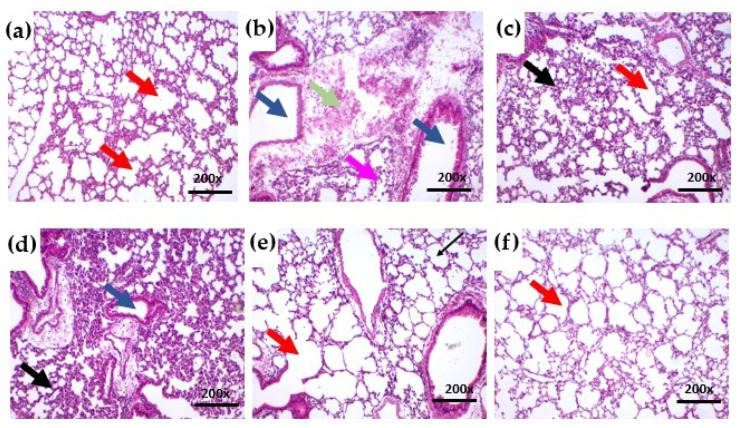
Histopathological analysis of the right lung from healthy (Sham) mice (**a**) as well as mice with LPS-induced acute lung inflammation previously treated intraperitoneally with PBS, control (**b**), dexamethasone at 2 mg/kg (**c**), or SteLL at 1 mg/kg (**d**), 5 mg/kg (**e**), or 10 mg/kg (**f**). Hematoxylin–eosin staining; magnification 200×. Key features: alveolar chambers (red arrows), bronchioles (blue arrows), congested vessels (green arrows), thickened epithelium (black arrows), and inflammatory infiltrate (pink arrows).

Histopathological analysis showed that lungs from the healthy (Sham) group had a regular epithelium of normal thickness and alveolar chambers of normal size, without edema or congestion (Figure 4a). In contrast, the control group exhibited massive infiltration of inflammatory cells throughout the pulmonary parenchyma, accompanied by venous congestion (Figure 4b). In the dexamethasone-treated group, a reduction in alveolar chambers was observed, suggesting a potential decrease in respiratory capacity (Figure 4c). The SteLL 1 mg/kg group also showed inflammatory signs, which were more pronounced and associated with epithelial thickening (Figure 4d).

Conversely, lungs from animals treated with SteLL at 5 and 10 mg/kg (Figure 4e,f) resembled those of the healthy group, displaying regular alveolar epithelium, preserved thickness, normal alveolar spaces, and reduced inflammatory infiltration compared with control. In the SteLL 10 mg/kg group (Figure 4f), erythrocytes were occasionally observed in some alveolar spaces; however, these represent minor changes likely due to transient vascular permeability induced by LPS instillation and do not reflect impaired lung function. Importantly, no structural alterations or significant tissue injury were detected, indicating that SteLL treatment substantially mitigated ALI while maintaining essentially normal pulmonary architecture.

## 3. Discussion

ALI is characterized by severe hypoxemic respiratory failure resulting from immune hyperactivation, oxidative stress, and cellular damage, leading to increased vascular permeability, pulmonary edema, and impaired gas exchange [40,41]. Despite advances in supportive care, current therapies have limited efficacy and safety, highlighting the need for novel interventions. In this study, pre-treatment with SteLL effectively mitigated key pathological features of LPS-induced ALI mice, including leukocyte and neutrophil infiltration, protein-rich edema, MPO activity, and pro-inflammatory cytokine release, while preserving lung architecture. Importantly, these parameters were comprehensively assessed in BALF, which directly reflects the local inflammatory milieu of the lung.

LPS is widely used in rodent ALI models because it induces an intense inflammatory response closely resembling human pathology. LPS administration disrupts the alveolar epithelium, promotes endothelial activation, upregulates adhesion molecules (P-selectin, E-selectin, ICAM-1, VCAM-1), and drives neutrophil recruitment. This cascade leads to barrier leakage, protein-rich edema, and amplification of inflammatory signaling through enzymes, reactive oxygen species, and cytokines [13,42,43]. SteLL treatment mitigated total leukocyte and neutrophil migration into BALF, suggesting attenuation of this inflammatory cascade. Lower MPO activity further indicates mitigation of neutrophil-mediated oxidative damage, contributing to preservation of alveolar-capillary integrity and limiting edema formation. By attenuating protein extravasation, SteLL can help maintain surfactant function, alveolar stability, lung compliance, and gas exchange [44,45]. Together, the findings demonstrate a consistent modulation of key local inflammatory processes central to ALI pathophysiology.

Notably, the cytokine panel analyzed provides a broad view of the inflammatory response modulated by SteLL within the pulmonary compartment. This anti-inflammatory effect is clinically relevant, as uncontrolled cytokine release, such as in COVID-19-associated cytokine storm, can drive severe pulmonary dysfunction and respiratory failure [46,47]. By dampening both neutrophil-mediated tissue damage and cytokine production, SteLL addresses central mechanisms underlying ALI. Histological analysis confirmed that higher doses of SteLL preserved alveolar architecture and limited inflammatory alterations, while lower doses showed mild epithelial thickening and residual inflammation.

The previously reported effects of SteLL in carrageenan-induced peritonitis [33] complement and reinforce its protective role observed in LPS-induced ALI. In both models, SteLL significantly reduced leukocyte migration, particularly neutrophil infiltration, and decreased markers of oxidative damage, such as MPO activity. Cytokine modulation was also consistent across models as SteLL limited excessive pro-inflammatory responses, including TNF-α. These results suggest a capacity to control immune activation while minimizing tissue injury, highlighting the broad immunomodulatory potential of SteLL. Taken together, these findings support that SteLL exerts anti-inflammatory effects across distinct inflammatory contexts, as evaluated in compartment-specific settings.

Unlike highly toxic lectins such as abrin, which cause severe lung injury, excessive inflammation, oxidative stress, and pulmonary edema even after a single inhalation exposure in mice [48], SteLL is a non-toxic lectin in rodents [33,34]. In the present work, pre-treatment of mice with SteLL did not induce acute toxicity or lung tissue damage, while effectively modulating neutrophil infiltration, cytokine release, and vascular permeability. It is important to note that absence of systemic inflammation can be inferred from the lack of clinical and histopathological alterations. This highlights that SteLL’s activity confers beneficial immunomodulatory and anti-inflammatory effects without the ribotoxicity or cytotoxicity observed with lectins like abrin [48].

The anti-inflammatory effect of lectins on lung inflammation models remains underexplored. However, the *Urtica dioica* agglutinin (UDA) demonstrated protection in a SARS-CoV mouse model, but this effect was attributed to direct antiviral activity [49] rather than modulation of host inflammation. While UDA inhibits viral adsorption and replication by binding viral glycoproteins, the beneficial effects of SteLL observed in ALI are anti-inflammatory and immunomodulatory, as SteLL has not yet been evaluated in infectious models.

The TIP peptide, which mimics the lectin-like domain of TNF, improved barrier integrity, enhanced alveolar fluid clearance, and reduced inflammatory cytokines and bacterial translocation in ex vivo human lungs [50]. TIP acts by directly modulating epithelial ion channels, suggesting avenues for future mechanistic investigations of SteLL.

The dose–response profile of SteLL in the preventive treatments analyzed suggests that its anti-inflammatory effects do not follow a simple linear relationship. When administered prior to ALI induction, the 5 mg/kg dose showed the most consistent effect in modulating the proportion of neutrophils and mononucleated cells, whereas lower (1 mg/kg) or higher (10 mg/kg) doses produced comparable effects in other parameters, indicating the presence of an optimal preventive therapeutic window. In contrast, control of plasma extravasation and MPO activity reached maximal effect even at the lowest preventive dose, suggesting a saturation of response. These results indicate that SteLL exhibits dose-dependent immunomodulatory activity in a preventive setting, but the relationship is not proportional, a pattern common for agents targeting complex inflammatory processes. Furthermore, the differences in dose–response profiles across the various parameters suggest that SteLL’s preventive effects likely involve distinct mechanisms or pathways for each outcome, reflecting the complexity and potentially independent regulation of leukocyte migration, cytokine production, and vascular permeability prior to ALI onset.

This work offers a new perspective on the development of natural products as potential therapeutics for ALI, addressing a research gap regarding the use of plant lectins in pulmonary inflammatory disorders. Nevertheless, as an initial investigation, further studies are required to fully elucidate the mechanisms through which lectins confer protection in ALI. Here, the anti-inflammatory phenotype of SteLL in ALI was demonstrated, paving the way for future research aiming at uncovering the underlying molecular mechanisms. This includes exploring canonical pathways such as Toll-like receptor 4/nuclear factor kappa B (TLR4/NF-κB) signaling, mitogen-activated protein kinase (MAPK) pathway, pyroptosis, and oxidative stress [51,52,53], to determine whether SteLL mediates its effects via direct inhibition of inflammatory signaling, modulation of immune cell activity, or alternative mechanisms.

As this study was specifically designed to investigate local pulmonary inflammation, systemic inflammatory responses were not evaluated. The use of BALF as the primary biological matrix is well established in ALI models, as it provides a direct and sensitive assessment of inflammatory processes occurring within the lung microenvironment. Nevertheless, given the recognized interplay between pulmonary and systemic inflammation, future studies should incorporate circulating biomarkers to further characterize the systemic impact of SteLL and strengthen translational interpretation.

Reductions in MPO activity and pro-inflammatory cytokines observed in BALF suggest that these molecules could serve as early indicators of therapeutic efficacy of SteLL. Compared to conventional anti-inflammatory therapies, SteLL offers the advantage of simultaneously reducing neutrophil-mediated damage, cytokine release, and vascular leakage, suggesting a multi-protective effect. These results warrant further preclinical and clinical investigations to evaluate SteLL as a potential adjunct therapy for ALI and ARDS.

While these findings are promising, extrapolation to human ALI requires caution. LPS-induced models do not fully replicate the complexity of clinical disease, including infectious triggers, heterogeneous lung injury, and comorbidities. In addition, further investigation is needed regarding the optimal administration routes and treatment windows before advancing. For instance, the impact of SteLL on both the acute and recovery phases of ALI and the potential of delivery strategies such as nebulization or intranasal administration need future exploration. Another potential strategy is the combination of SteLL with established drugs, such as dexamethasone, given that combination therapy represents an important avenue in clinical drug development. Also, studies should also evaluate the effects of SteLL in infectious models of ALI to enhance translational relevance.

One more important consideration for clinical translation of lectins is their immunogenicity and immunomodulatory profile. Lectins, as proteins, can elicit immune recognition due to their amino acid sequences, three-dimensional structures, or post-translational modifications, potentially stimulating antibody production or inflammatory responses [54]. SteLL has been shown to induce mice splenocytes to secrete both pro-inflammatory cytokines (IL-17A, TNF-α, IFN-γ, IL-2) and anti-inflammatory IL-4, suggesting a capacity to modulate immune responses without triggering excessive inflammation [32]. In both peritonitis and ALI models, SteLL increased anti-inflammatory cytokines while limiting pro-inflammatory mediators, highlighting an advantage over highly immunogenic or purely pro-inflammatory lectins. However, careful evaluation of immune responses, including antibody generation and systemic effects, will be critical in future preclinical and clinical studies. Strategies such as controlled dosing, nanoparticle or liposome formulations, and targeted delivery can minimize undesired immunogenicity while preserving lectins therapeutic benefits [54].

## 4. Materials and Methods

### 4.1. SteLL Purification

*S. terebinthifolia* leaves were collected at the campus of the *Universidade Federal de Pernambuco* (UFPE) (Recife, Pernambuco, Brazil), with authorization (nº 72024) from the *Instituto Chico Mendes de Conservação da Biodiversidade* (ICMBio). The study was recorded under code AED6BF8 in the *Sistema Nacional de Gestão do Patrimônio Genético e do Conhecimento Tradicional Associado* (SisGen). A representative specimen of the species was deposited at the *Instituto Agronômico de Pernambuco* (IPA, Recife) under the reference number 73431.

SteLL was purified following the methodology from Gomes et al. [24]. The collected leaves were first rinsed under running water and then with distilled water before being air-dried at 28 °C for 4 days. Once dried, the leaves were ground into a fine powder using an industrial processor (LQL-4, Metvisa, Brusque, Brazil). For extract preparation, the powdered leaves were suspended in 0.15 M NaCl at a proportion of 10% (*w*/*v*) and homogenized for 16 h at 4 °C. The homogenate was then centrifuged at 9000× *g* for 15 min at 4 °C. The resulting leaf extract, containing 15 mg/mL of protein, was applied (2 mL) to a chitin column (7.5 × 1.5 cm; Sigma-Aldrich, St. Louis, MO, USA) pre-equilibrated with 0.15 M NaCl. Non-binding proteins were removed through washing, and SteLL was subsequently eluted using 1.0 M acetic acid. The elution was monitored by measuring absorbance at 280 nm. The purified lectin was dialyzed against distilled water for roughly 6 h using a cellulose membrane with a 10 kDa cut-off (Sigma-Aldrich), followed by lyophilization. Finally, SteLL was resuspended in PBS and stored at low temperature until further use.

### 4.2. Determination of Protein Concentration and Hemagglutinating Activity

Protein concentration was quantified using the Lowry et al. [55] method, employing a bovine serum albumin (Sigma-Aldrich) standard curve ranging from 31.25 to 500 μg/mL. The carbohydrate-binding activity of SteLL was evaluated through the hemagglutination assay. A 50 μL aliquot of the SteLL sample was subjected to successive twofold serial dilutions in 0.15 M NaCl across the wells of a 96-well microplate. Subsequently, 50 μL of a suspension of New Zealand rabbit erythrocytes, fixed with glutaraldehyde [56], were added to each well. Erythrocytes incubated exclusively with 0.15 M NaCl served as control. All assays were carried out in duplicate. The hemagglutinating activity was defined as the reciprocal of the highest lectin dilution capable of producing complete agglutination of the erythrocytes. Specific hemagglutinating activity was calculated by dividing the hemagglutinating activity by the protein concentration (mg/mL).

### 4.3. Mice

Forty-eight female BALB/c mice aged 6–8 weeks and weighing 20–25 g were obtained from the animal facility of the *Instituto Keizo Asami* (iLIKA) of UFPE. After arrival, the animals were acclimated and maintained at the *Laboratório de Experimentação Animal em Bioquímica* (LEAB), Department of Biochemistry, UFPE. The mice were kept under controlled environmental conditions, including a temperature of 22 °C, a 12 h light–dark cycle, and free access to food and water.

### 4.4. Treatments and LPS-Induced ALI

The mice were randomly assigned to six experimental groups using a simple randomization method, with eight animals per group (*n* = 8) maintained in the same cage. The chosen sample size was considered sufficient to detect biologically and statistically meaningful differences between groups while maintaining ethical use by avoiding the unnecessary use of experimental animals. To control for potential confounding variables, the experimental treatments were performed in a fixed order: Sham, negative control, SteLL-treated groups with escalating doses, followed by dexamethasone. Knowledge of group allocation was restricted to the researcher responsible for administering the treatments at each stage. Throughout the experiment, none of the animals exhibited signs of toxicity indicative of humane endpoints. Thus, there was no exclusion of animals.

Treatments were administered by intraperitoneal injection of SteLL at doses of 1, 5, or 10 mg/kg, dexamethasone (2 mg/kg), or PBS as the vehicle (control). One hour later, anesthesia was induced using ketamine (100 mg/kg, i.p.) and xylazine (10 mg/kg, i.p.), followed by induction of ALI via intranasal instillation of 25 μL of LPS (1 μg/µL; *Escherichia coli*, serotype 026:B6; Sigma-Aldrich) [57]. A healthy control group (Sham) received 25 μL of PBS intranasally and was pre-treated with the vehicle only. Animals were euthanized (300 mg/kg ketamine and 30 mg/kg xylazine i.p.) 24 h post-ALI induction. BALF was collected using 1 mL of PBS containing 1 mM ethylenediaminetetraacetic acid. The right lungs were then excised, weighed, and processed for histopathological examination.

### 4.5. BALF Analysis

#### 4.5.1. Cell Count

BALF samples were centrifuged at 3000× *g* for 5 min at 25 °C. The resulting cell pellets were resuspended in Turk’s solution (1:20 dilution), and total leukocyte counts were performed using a Neubauer chamber (Kasvi, Pinhais, Brazil) under an optical microscope (Eclipse E100, Nikon, Tokyo, Japan). Results were expressed as the total number of cells per milliliter of BALF (leukocytes/mL). For differential cell counts, smears were prepared, stained with Papanicolaou’s stain, and 100 leukocytes per slide were classified under optical microscopy. Data were presented as the percentage of neutrophils and mononucleated cells.

#### 4.5.2. Measurement of Inflammatory Markers

##### Total Protein Concentration

The total protein concentration in the BALF supernatant (1 mL) was measured using the Bicinchoninic Acid (BCA) Protein Assay Kit (Sigma-Aldrich) according to the manufacturer’s instructions. Protein levels were quantified in μg/mL based on a standard curve generated with bovine serum albumin.

##### Measurement of Cytokine Release

Cytokine levels in the BALF supernatant were measured using the Cytometric Bead Array (CBA) Mouse Th1/Th2 Cytokine Kit II (BD Biosciences, Franklin Lakes, NJ, USA), enabling detection of IFN-γ, IL-2, IL-4, IL-6, IL-10, IL-17, and TNF-α, according to the manufacturer’s protocol. Samples were analyzed on a BD Accuri C6 flow cytometer (BD Biosciences). Individual standard curves ranging from 0 to 5000 pg/mL were generated, and cytokine concentrations were calculated using BD Accuri C6 software 264.21.

##### NO Production

NO production in the BALF supernatant was estimated by measuring nitrite levels using the Griess assay, following the method described by Ding et al. [58]. Nitrite concentrations were determined based on a standard curve of sodium nitrite ranging from 3.12 to 400.00 μM.

##### MPO Activity

MPO activity was assessed following the method of Bradley et al. [59] with minor modifications. Briefly, 20 μL of BALF supernatant was added to a 96-well microplate, and the reaction was initiated by adding 180 μL of o-dianisidine (Sigma-Aldrich) solution (0.167 mg/mL in phosphate buffer, pH 6.0) with 1% hydrogen peroxide (H_2_O_2_) as the substrate. After 15 min of incubation, absorbance was measured at 450 nm using a microplate spectrophotometer (Epoch, BioTek Instruments, Inc., Winooski, VT, USA), with the resulting values reflecting MPO enzymatic activity.

### 4.6. Histological Analysis of Lung Tissue

The right lung was excised, and 5 μm tissue sections were fixed in 10% buffered formalin. The samples were then embedded in paraffin and stained with hematoxylin and eosin. Histological analysis was performed using a DM500 brightfield microscope (Leica, Wetzlar, Germany) equipped with a ICC50W camera (Leica), at 100× and 200× magnifications, to assess the cellular inflammatory response.

### 4.7. Statistical Analysis

Data analysis was performed using GraphPad Prism^®^ version 8.0 (GraphPad Software, San Diego, CA, USA). Results are expressed as the mean ± standard error of the mean (SEM). Normality of the data was evaluated using the Shapiro–Wilk test. Comparisons between experimental groups were conducted using one-way analysis of variance (ANOVA) followed by Tukey’s post hoc test. Differences were considered statistically significant at *p* ≤ 0.05. With regard to cytokine quantification, data were analyzed using one-way ANOVA followed by Tukey’s post hoc test, with *p*-values adjusted using the Benjamini–Hochberg false discovery rate (FDR) method, yielding adjusted q-values ranging from 0.0001 to 0.0007.

## 5. Conclusions

SteLL attenuated key features of ALI induced by lipopolysaccharide, including leukocyte infiltration, inflammatory mediator production, and lung tissue damage. These findings reinforce its anti-inflammatory activity in the pulmonary microenvironment. Coupled with its low acute toxicity at the doses tested, the results encourage further research to evaluate systemic effects, effectiveness across both sexes, pharmacokinetic properties, and the molecular mechanisms responsible for its anti-inflammatory effects. Such studies will be essential to better define the translational potential of SteLL.

## Data Availability

The original contributions presented in this study are included in the article. Further inquiries can be directed to the corresponding author.
